# Evaluation of outcomes of psychological interventions in terminally ill family caregivers: a brief overview

**DOI:** 10.3389/or.2024.1482195

**Published:** 2024-11-26

**Authors:** Masoud Rezaei, Sahar Keyvanloo Shahrestanaki, Arezoo Sheikh Milani, Masoumeh Neishabouri, Shiva Khaleghparast, Mohammad Reza Rajabi

**Affiliations:** ^1^ Nursing and Midwifery Care Research Center, School of Nursing and Midwifery, Iran University of Medical Sciences, Tehran, Iran; ^2^ Cardiovascular Nursing Research Center, Rajaie Cardiovascular Medical and Research Center, Iran University of Medical Sciences, Tehran, Iran; ^3^ Department of Community Health Nursing and Geriatric Nursing, School of Nursing and Midwifery, Tehran University of Medical Sciences, Tehran, Iran; ^4^ Department of Community Health Nursing, School of Nursing and Midwifery, Shahid Beheshti University of Medical Sciences, Tehran, Iran; ^5^ Nursing and Midwifery Care Research Center, Health Management Research Institute, Iran University of Medical Sciences, Tehran, Iran; ^6^ Department of Cardiology, School of Medicine, Shahed University, Tehran, Iran

**Keywords:** cancer, family caregiver, psychological interventions, advanced cancer caregivers, end of life

## Abstract

**Background:**

Family caregivers play a crucial role in supporting patients with incurable diseases, but often experience significant stress and anxiety. This study aimed to investigate the impact of psychological interventions on family caregivers, with a focus on reducing the burden of care, improving mental health and quality of life, and promoting family communication.

**Method:**

This study conducted a brief overview of quantitative and qualitative research on assessing the impact of psychological interventions on family caregivers. A comprehensive literature search was conducted in PUBMED, SCOPUS, EMBASE, SCIENCE DIRECT and WEB OF SCIENCE to identify relevant papers, resulting in 20 articles being included. The included studies focused on evaluating the outcomes of psychological interventions on family caregivers.

**Result:**

Ultimately, 20 relevant articles were retrieved from a pool of 500 articles, focusing on the evaluation of the primary outcomes of psychological interventions on family caregivers. The review of 20 articles revealed that interventions such as expressive writing and reminiscence therapy had a positive and significant impact on reducing the burden of care and fostering a positive care environment. Additionally, these interventions were found to be effective in improving self-esteem, family communication, and overall wellbeing.

**Conclusion:**

The study emphasizes the need for further research to confirm the benefits of these interventions and their role in promoting family resilience. These findings highlight the potential of psychological interventions in alleviating the challenges faced by family caregivers of patients with incurable diseases.

## Introduction

Nowadays, with the rise in incurable diseases and limited access to advanced home care facilities, there is a growing dependence on family caregivers to provide for patients with advanced illnesses (1). When a family member is diagnosed with an incurable disease, the individual’s perception of life shifts towards living with the symptoms of the disease. This alteration impacts not just the patient, but also their loved ones and other family members. As a result, caregivers of patients require various care services (2).

Family caregivers are defined as family members, friends, or individuals with a close relationship to the patient who provide care (3). At the time of diagnosis, family caregivers accompany patients to medical appointments, become the patients’ advocates, the primary decision makers on patients’ behalf, provide emotional support, and companionship, in addition to executing medical plans without prior preparation or experience (4). In the United States, at least 12 million people care for their loved ones at home, often sacrificing personal activities such as attending school, going to work, or self-care (5). Family caregivers play a vital role in assisting with daily tasks, coordinating with medical staff, and providing care. Spouses of terminally ill patients dedicate over 100 h per week to these responsibilities (6–8). Unfortunately, there is substantial evidence documenting deleterious caregiver burden. As people live longer with advanced illnesses, the negative effects of caregiver burden will only continue to grow (9). A meta-analysis of 84 caregiver burden studies across all types of chronic disease showed that caregivers exhibit higher levels of stress and depression, lower well-being, and worse physical health compared to non-caregiver controls (10). The results of numerous studies show that the possibility of developing cardiovascular diseases, immunodeficiency, diabetes, and mortality is higher in caregivers of incurable patients (11–13).

Various psychological interventions have been conducted to support caregivers in facing their challenges. These interventions focus on social and psychological factors rather than biological factors and aim to enhance quality of life, social functioning, and symptom improvement in individuals (14). Examples of psychological interventions include family-based dignity therapy, expressive writing (15), life review intervention (14), narrative therapy, and reminiscence therapy (16, 17). Seyed Fatemi et al.'s study (2021) suggests that psychological interventions like dignity therapy and expressive writing may enhance caregivers’ self-awareness and ability to cope with caregiving issues (15). Kleijn et al. (2018) found that life review intervention does not reduce depression and anxiety symptoms in caregivers of cancer patients but does improve their self-esteem (14). Due to limitations in existing research, there is a need for a comprehensive study to confirm the impact of these interventions on family caregivers of patients with incurable diseases. Therefore, this study aims to investigate the effect of psychological interventions on family caregivers of incurable patients.

## Methods/approach

### Study design

This study conducted a brief overview of quantitative and qualitative research on assessing the impact of psychological interventions on family caregivers. A brief review delves into a specific aspect of information within a larger article or the entire article. Its aim is to offer an overview of current knowledge on a topic, and/or to set the stage for new research (1). This concise review study encompassed a summary of prior findings in the literature regarding the assessment of the primary effects of psychological interventions on family caregivers. The author (ASHM) conducted a search for articles, and the extracted documents were then independently reviewed by other researchers to ensure the inclusion of relevant and appropriate materials in the research. The review followed PICO (Terminally Ill Family Caregivers (Population), how effective are Narrative Psychological Interventions (Intervention), Comparison (Narrative Psychological Interventions comparison) in Psychological Outcomes (Outcome)) instruction in conducting this study. Initially, a team of doctoral students and nursing faculty members from Iran University of Medical Sciences and Rajaie Cardiovascular Medical and Research Center was established.

### Search strategies

**FIGURE 1:**
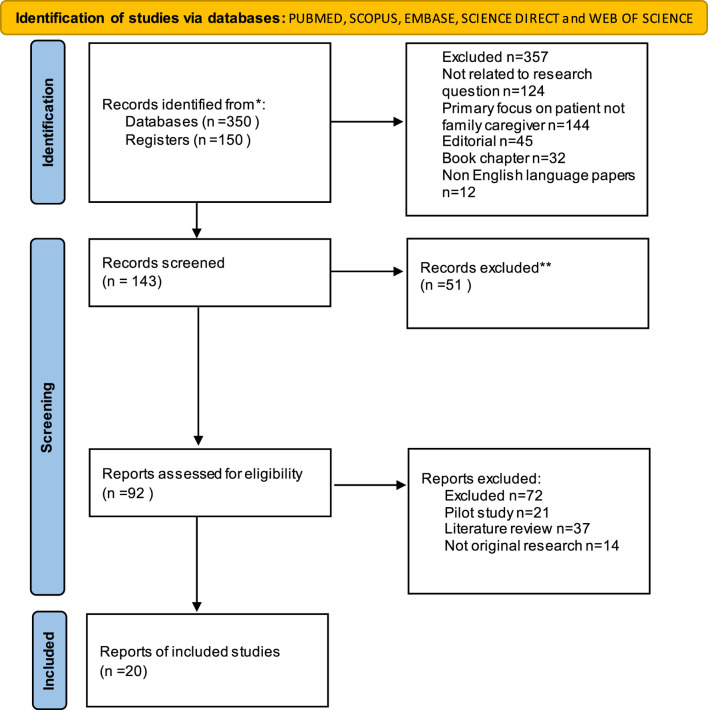
PRISMA flow diagram.

Searching electronic databases such as SID, IRANMEDEX, MAGIRAN, Google Scholar, PUBMED, SCOPUS, and Web of Science in both English and Farsi from 2000 to 2023, using keywords such as family caregivers, care, family, psychological interventions, life review intervention, reminiscence therapy, dignity therapy, narrative therapy, expressive writing, and palliative care, was conducted. Ultimately, 20 relevant articles (Figure 1) were retrieved from a pool of 500 articles, focusing on the evaluation of the primary outcomes of psychological interventions on family caregivers. MeSH-related terms and keywords were reviewed to confirm that the selected articles contained appropriate search terms. The search strategy in PubMed was: “Evaluation Outcomes” OR “Study Outcome” OR “Outcome Assessment” AND “Psychological Interventions” OR Psychotherapy OR “Psychological Care” AND “Terminally Ill” OR “Advanced Cancer” OR “End of Life Cancer” AND “Family Caregiver” OR “Informal Caregiver.” Studies with relevant titles and abstracts were analyzed. The retrieved articles were screened based on inclusion and exclusion criteria.

### Eligibility criteria

Inclusion criteria for this review included a wide range of quantitative and qualitative studies that explicitly evaluated primary outcomes of psychological interventions aimed at family caregivers of patients receiving palliative care. These interventions are critical because they address the unique challenges that caregivers face during this period, both emotionally and physically. The psychological outcomes of interest are various psychological variables identified in previous research, including, but not limited to, enhancing caregivers’ sense of meaning and purpose in their roles, enhancing their coping skills, and reducing negativity, mental states such as anxiety, stress, and burnout. These outcomes are important because they not only improve the wellbeing of caregivers but also affect the quality of care provided to patients. Focusing on these aspects, this review aims to highlight the importance of psychological support interventions in enhancing family caregivers’ overall experience of palliative care. On the other hand, the exclusion criteria were carefully defined to maintain the integrity and focus of the analysis. Specifically, we excluded editorial sections, book chapters, pilot studies, and any research that focused primarily on patients themselves rather than family caregivers. This refined approach ensures that the literature reviewed is directly related to the psychological needs and experiences of caregivers, allowing for a clearer understanding of the effectiveness of interventions designed to support them. By creating these criteria, we aim to create a targeted review that truly reflects the challenges that caregivers face and the interventions that can meaningfully support them in their critical role. Subsequently, the pertinent articles underwent critical evaluation, encompassing methodological and result-related assessments to identify strengths, weaknesses, and their alignment with the study’s review objective. The primary focus was on assessing the impact of psychological interventions on family caregivers. All retrieved articles were thoroughly read by one author and then reviewed by a second author to ensure a comprehensive understanding of the studies. Three authors (MR, SKSH, and SHKH) utilized standard data collection tools to extract data. Primary study authors were contacted for additional information as needed. Data extraction was based on publication date. The main characteristics of the analysis included (a) whether the study specifically focused on evaluating the outcomes of psychological interventions on family caregivers; (b) whether the study was conducted for family caregivers, families, or both; (c) methods; (d) content; and (e) results (evaluation of the main consequences of psychological interventions). Based on the common meanings and key themes of the findings, we attempted to expand the definition of the primary outcome in further analyses and evaluate the main outcomes of psychological interventions.

## Results

### Characteristic of the included studies

Ultimately, 20 relevant articles (Figure 1) were retrieved from a pool of 500 articles, focusing on the evaluation of the primary outcomes of psychological interventions on family caregivers.All studies were written in English. Studies have been conducted in United States, Canada, Australia, Iceland, Nederland, china, India, Italy, japan and Iran. Relative caregivers to patient was spouse, husband, partner, parents, brother, sister and children. These studies (two quasi-experimental, five randomized controlled trials, two pre/post intervention, one mixed-methods, five experimental studies and six qualitative) were included in this review and was summarized in the Table 1.

**TABLE 1:** Eligible studies.

Number	Title	Intervention	Country (Year)	Study type (study design)	Questionnaire	Main intervention Consequence(s)
1	Bereavement life review improves spiritual wellbeing and ameliorates depression among American caregivers (18)	Life review intervention	Hawai-America (2015)	Quasi-experimental	- Functional Assessment Chronic Illness Therapy–Spiritual (FACIT–Sp) questionnaire - Beck Depression Inventory, Second Edition (BDI-II)	Efficacy for spiritual wellbeing and depression
2	A randomized controlled trial on the efficacy of life review therapy targeting incurably ill cancer patients: do their informal caregivers benefit? (8)	Life review intervention	Netherlands (2021)	Randomized controlled trial	- Caregivers reaction assessment scale (CRA) - Hospital anxiety and depression scale - Post traumatic growth inventory	No significant effect was found on these symptoms nor on posttraumatic growth or most aspects of caregiver burden. There was a significant effect of Life Review Therapy and Memory Specificity Training (LRT-MST) on the course of self-esteem
3	Acceptability of a Telehealth Dyadic Life Review Intervention for Older Adults with Advanced Cancer and Their Caregivers (7)	Life review intervention	Michigan, United States (2023)	Pre/post intervention design	FOCUS Satisfaction Instrument	A majority of individuals with cancer and their caregivers reported that the program helped them cope with cancer (individuals with cancer = 86%, caregivers = 81%). Almost all participants reported that they would recommend the program to others facing cancer
4	Effect of reminiscence therapy based on positive psychology theory (RTBPPT) on the positive feelings of the spousal caregivers of elderly patients with advanced cancer in China (19)	Reminiscence therapy	China (2020)	Randomized controlled trial	- Personal Information Form - The Zarit Burden Interview (ZBI) - Positive Aspects of Caregiving (PAC) instrument - Herth Hope Index (HHI)	Reduction in the burden of spousal care, higher levels of positive feelings and hope; Reminiscence therapy is an effective approach to reducing the care burden of spouses of elderly patients with advanced cancer and enhancing the spouses’ positive feelings and hope
5	Development and evaluation of the Dignity Talk question framework for palliative patients and their families A mixed-methods study (20)	Dignity therapy	Manitoba, Canada (2018)	convergent parallel mixed-methods design	Open-ended Dignity Talk questions	Participants felt Dignity Talk would be valuable in promoting conversations, enhancing family connections and relationships, enhancing patient sense of value and dignity promoting effective interaction, and attending to unfinished business. Participants suggested that patients and family members be given latitude to respond only to questions that are meaningful to them and within their emotional capacity to broach
6	Dignity therapy for people with motor neuron disease and their family caregivers: a feasibility study (12)	Dignity therapy	Australia (2015)	Cross-sectional using a single intervention group and repeated measures pretesting and posttesting	- Patient Dignity Inventory (PDI) - Amyotrophic Lateral Sclerosis Assessment Questionnaire-5 (ALSAQ-5) - Functional Assessment of Chronic Illness Therapy-The 12-item Spiritual Wellbeing Scale (FACIT-sp 12) - Herth Hope Index (HHI) - Zarit Burden Interview (ZBI-12) - The Hospital Anxiety and Depression Scale (HADS)	Family caregivers overwhelmingly agreed that the dignity therapy document is and will continue to be a source of comfort to them and they would recommend dignity therapy to others in the same situation
7	Dignity therapy: a feasibility study of elders in long-term care (13)	Dignity therapy	Manitoba, Canada (2012)	Experimental study	- Mini Mental Status Examination (MMSE) - Dignity Therapy Question Protocol Modified feedback questionnaire containing questions such as: where is the dignity therapy manuscript kept; who has read it; how often it has been read; what influence family members feel it has had on their relative; what influence family members feel it may have had on care providers; and what influence it has had on them, personally	The majority of proxy participants indicated that dignity therapy would be helpful to them and their families. In both groups, HCPs reported the benefits of dignity therapy in terms of changing the way they perceived the resident, teaching them things about the resident they did not previously know; the vast majority indicated that they would recommend it for other residents and their families
8	Translating dignity therapy into practice: effects and lessons learned (14)	Dignity therapy	India (2013)	Experimental study	- 21-item Beck Depression Inventory, 2nd edition - 46-item Functional Assessment of Chronic Illness Therapy-Palliative Care (FACIT-Pal) scale anxiety, sense of suffering desire for hastened death, lack of wellbeing, and perceived loss of dignity were assessed with five single items rated on a 7-point ordinal scale (0 = not a source of distress, 1 = minimal distress, 2 = mild distress, 3 = moderate distress, 4 = strong distress, 5 = severe distress, and 6 = extreme distress)	100% of family respondents (n = 6) said reading the legacy book was helpful to them. Seventy-five percent of patients agreed that DT made their life more meaningful, while 100% of families indicated that they derived meaning from reading the legacy document
9	Effect of dignity therapy on distress and end-of-life experience in terminally ill patients: a randomized controlled trial (15)	Dignity therapy	Winnipeg, New York, Perth (2011)	Multi-site randomized controlled trial	- FACIT Spiritual Wellbeing Scale - Patient Dignity Inventory, the Hospital Anxiety and Depression Scale - Quality of Life Scale Modified Edmonton Symptom Assessment Scale	Dignity Therapy was significantly more likely to be experienced as helpful, improve quality of life, sense of dignity; change how their family sees and appreciates them and be helpful to their family
10	Effects of family-based dignity intervention and expressive writing on anticipatory grief in family caregivers of patients with cancer: a randomized controlled trial (17)	Dignity therapy	Iran (2023)	Randomized Controlled Trial	13-item anticipatory grief scale (AGS)	a significant reducing effect of family-based dignity intervention on AGS and its subscales including behavioral compared with the control group
11	Striving to thrive: A qualitative study on fostering a relational perspective through narrative therapy in couples facing cancer (6)	Narrative therapy	United States (2020)	Qualitative study with a multiple case study design and thematic analysis	------------------	Participants learned to be vulnerable, increased mindfulness, and improved communication
12	The power of informal cancer caregivers’ writings: results from a thematic and narrative analysis (21)	Expressive writing	Italy (2021)	A qualitative study	--------------	Expressive writing benefit informal caregivers (ICs) with respect to the possibility of sharing their experiences with others and giving evidence of their role. Their stories are a testimony that can help those who face a similar experience
13	Cocreating Meaning Through Expressive Writing and Reading for Cancer Caregivers (22)	Expressive writing	Canada (2019)	Experimental study	--------------	Expressive writing and reading can be a safe and cost-effective supportive intervention for caregivers of patients with cancer. Perceived benefits of expressive writing and reading included emotional and cognitive processing (individual and collaborative), learning from the emotions and experiences of other caregivers,and preparing for upcoming challenges
14	The impact of written emotional disclosure on cancer caregivers’ perceptions of burden, stress, and depression: A randomized controlled trial (3)	Expressive writing	United States (2017)	A Randomized Controlled Trial	- The 10-item Perceived Stress Scale - 22-item Caregiver Burden Scale - The Patient Health Questionnaire	Improve burden, stress, and depression, positive framing condition
15	Expressive Writing as a Coping Mechanism for Caregivers of People with Parkinson’s Disease (4)	Expressive writing	United States (2016)	A Qualitative study	- Caregiver Burden Inventory - Brief Cope - Brunel Mood Scale - Instrumental Activities of Daily Living	Expressive writing was most beneficial for participants caring for loved ones in the earlier stages of Parkinson’s and placed in the positive framing condition
16	Writing Therapy for the Bereaved: Evaluation of an Intervention (23)	Expressive writing	Australia (2003)	Experimental study	Grief and General Health Questionnaire-30 (GHQ-30)	Writing therapy offers a useful, cost-effective, and private way of supporting bereaved individuals who may not practice self-care
17	Using narrative approach for anticipatory grief among family caregivers at home (24)	Expressive writing	Japan (2016)	Qualitative intervention study	-------------	The narrative approach was effective in freeing family caregivers from the feeling of being trapped in their caregiver role
18	Caring for Caregivers: Assessing the Influence of Expressive Writing on Cance Caregivers’ Emotional Wellbeing, Relational Satisfaction, and Comforting Sensitivity (25)	Expressive writing	United States (2015)	Qualitative study	- The Caregiver Burden Scale - Perceived Stress Scale - Patient Health Questionnaire - Brief Resilience Scale - Multidimensional Scale of Perceived Social Support Scale - Modified version of the Investment Model Scale - Difficulty in Emotion Regulation questionnaire - Interactional Reactivity Index - Verbal person-centered support quality	support quality (measured via naturally-occurring support situations) may be improved upon through the use of particular ways of writing. Results also suggest that aspects of caregiver wellbeing may be positively, and sometimes negatively, affected by expressive writing
19	Treatment decision-making, family influences, and cultural influences of Chinese breast cancer survivors: a qualitative study using an expressive writing method (26)	Expressive writing	China (2020)	A phenomenological research method	-------------	Family influences included the subthemes of financial burden, family expectations, and family support
20	Does Expressive Writing Reduce Stress and Improve Health for Family Caregivers of Older Adults? (27)	Expressive writing	Canada (2007)	Experimental study	- Demographic questionnaire - Manipulation Check Questionnaire - The short form of the Zarit Burden Interview −15-item Impact of Events Scale −28-item General HealthQuestionnaire	Contrary to expectations, expressive-writing and history-writing participants performed similarly across outcomes. Only caregiver participants in the time-management condition experienced significant mental and physical health improvements after writing

### Impact of psychological intervention on family caregivers

The findings of studies reveal the impact of psychological interventions on family caregivers. There were four studies on Life review intervention, two on Reminiscence therapy, five on Dignity therapy, one on Narrative therapy, and nine on Expressive writing.

After completing the review process, the data related to the review question was extracted in the form of study subsets. These sub-categories were re-examined by members of the research team to describe the results in terms of three new main categories. These categories are provided in Table 2.

1 - Reducing the Burden of Care and Facilitating the Caregiving Process

**TABLE 2:** Main categories and related subcategories of study.

Related articles	Sub- categories	Main categories
5,6,18,19	• Reduction of care burden • Positive framing condition	Reducing the care burden and facilitating the care process
1–7,9–15,17,18,20–22,24	• Spiritual wellbeing • Self-Esteem • Higher family support • Coping with cancer • Positive feelings and hope Satisfaction • Promotion • Life more meaningful • Sense of dignity • Increased mindfulness • Improve stress and depression • Self-care • Freeing • Wellbeing • Management • Significant mental and physical health • Improvements	Improving mental health and quality of life
3-7-8-12–14-16–23	• Higher expressive family functioning • Emotional communication • Developed roles • Promoting conversations • Enhancing family connections and relationships • Change how their family sees and appreciates them • Improved communication • Sharing their experiences with others • Family expectations	Promotion of family communication

Based on the findings presented in Table 1, the literature review demonstrates that when it comes to caring for cancer patients and their families, methods such as expressive writing and reminiscence therapy have been found to have a positive and significant impact in reducing the burden of care for family caregivers and creating a spiritual environment. The results indicate that reminiscence therapy is more effective in reducing the burden of care (1, 2), while expressive writing is more helpful in creating a positive and hopeful care environment (17, 28). These methods, in addition to reducing stress in family caregivers, accompany them in creating hope for life and a more intimate relationship with each other. It was also shown in studies that these effects are not only on family caregivers and cancer patients also benefit from the positive and hopeful atmosphere resulting from these treatments (3). The impact of such interventions in other chronic diseases has also been able to create similar results, so that Beck et al.'s study reported that Expressive writing can be helpful in creating an optimistic and positive atmosphere in the early stages of Parkinson’s diagnosis (4).

2 - Improving Mental Health and Quality of Life

Life Review Therapy is known as an art therapy method that is used to help people reexamine their memories and life experiences, better understand themselves and structure their future. This method involves periodic sessions where the individual works with the help of a therapist to improve self-awareness as well as family relationships (5). The results showed that regarding the effects of this method on family caregivers of patients, this method can be effective in improving self-esteem and family communication (6). Also, many participants stated that this method has helped in the care of cancer patients and they recommend that others use this method as well (7).

By examining various studies related to the effects of Life Review Therapy on family caregivers of patients, interesting results have been obtained. The effect of this method on spiritual health and depression has not been significantly affected; Also, it did not have much effect on post-traumatic growth and the care burden of most aspects (8). However, a significant effect on self-esteem was observed (8). Also, more family support, more family performance in expressing emotions and emotional communication, and higher emotional dialogue increased significantly (21). Most participants with cancer and their family caregivers reported that Life Review Therapy helped them cope with cancer (participants with cancer = 86%, family caregivers = 81%). Almost all participants stated that they would recommend the program to others facing cancer (7).Studies conducted among caregivers of cancer patients show that the use of Life Review Therapy can be effective in improving self-esteem and family communication and help caregivers and cancer patients to cope with the disease (9, 10). On the other hand, the results showed that Life Review Therapy can be more effective on spiritual wellbeing and emotional aspects (21).

Other studies have shown that the effect of memory therapy on family caregivers of patients has increased the level of positive emotions and hope in addition to reducing the burden of tolerance in caring for the spouse. It seems that memory therapy has been an effective approach to reduce the burden of caring for the spouses of elderly patients with advanced cancer and has caused an increase in positive feelings and hope in the family caregivers who were the spouses of the patients (14). However, based on some studies, it was seen that the use of memory therapy did not have a long-term effect on non-family caregivers living in nursing homes (11).

In reviewing the studies, it was seen that Dignity Therapy (DT) has very positive effects on family caregivers of patients. The results of these studies have shown that the majority of family caregivers firmly stated that a DT document is a source of comfort for them and they guide others in the same situation to benefit from DT (12–15). The majority of participants indicated that DT would benefit them and their families. In addition, most family caregivers stated that DT has made their lives more valuable and spiritual, and this indicates the positive effect of DT on spiritual wellbeing in family caregivers (14, 16). Dignity Therapy significantly improves the quality of life and respect for patients’ dignity and improves their attitude and value in the eyes of the family (16, 17). Also, studies have shown that interventions based on family dignity have a significant effect on reducing behavioral symptoms (depression and anxiety) in family caregivers (17).

The results of the studies show that using Narrative Therapy has very positive effects on family caregivers. In these reviewed studies, family caregivers learned to experience openness and ease, greater awareness, and improved communication (6). The use of narrative therapy sessions in the care practice helps caregivers and gives them the opportunity to understand the pain and suffering of patients, listen to them, confirm them and share their feelings with them (28).

An effective way to help family caregivers manage stress and anxiety is to use the Express Writing technique. The results of a study have confirmed that people who regularly write and reread about their experiences and feelings experience a significant improvement in their depression, stress and anxiety (3). Also, this activity helps them, as caregivers, to better understand that others are facing the same problems and challenges that they are facing. This process allows them to benefit from the experiences of other caregivers of cancer patients and discover new ways to manage their emotions (3, 22). As a result, using this writing method can be effective as a simple and effective solution in improving family relationships and reducing the stress and anxiety of family caregivers.

The results of the studies show that using Expressive writing is a useful, low-cost and private method that is very helpful in supporting family caregivers. Expressive writing helps create a sense of freedom in family caregivers and frees them from feeling trapped in the caregiver role. In addition, it increases the quality of support for patients (24, 23, 25). Also, the results of a study showed that in what was expected, participants in Expressive writing experienced a significant improvement in mental and physical health after writing, and this strengthened their physical wellbeing (27).

3 - Promotion of Family Communication

In general, research shows that most psychological interventions such as: Life review intervention, Reminiscence therapy, Dignity therapy, Narrative therapy and Expressive writing can significantly improve the family communication of family caregivers of patients (21). The results showed that more support from the family had a significant improvement in the caring performance of the caregivers and a significant increase in emotional communication between them. In addition, interventions involving the use of memory-evoking techniques have also had a positive effect on family communication (6, 15, 16, 21). These researches show that according to appropriate psychological interventions, it is possible to make a significant improvement in family communication for family caregivers of patients (18). From the results, it can be concluded that due to the emotional tension and burden of care that families bear, their participation in psychological interventions can be useful for them.

## Conclusion

This study discusses various psychological interventions that have a positive effect on the wellbeing of family caregivers. Among these interventions, memory therapy, dignity therapy, life review therapy, narrative therapy, and expressive writing therapy can be mentioned. These treatments help caregivers cope with the emotional and psychological stress associated with caring for a patient who is very dear to them and who has an incurable and difficult disease. This article provides an overview of each psychotherapy, its benefits, and its impact on caregivers’ wellbeing. This article also emphasizes the importance of addressing the needs of caregivers and the need for more research in this area. This article emphasizes the need to further investigate the effect of these treatments on the well-being of caregivers and their role in promoting communication and family resilience. This article provides an overview of each psychotherapy, its benefits, and its impact on improving self-esteem, family communication, and overall caregivers’ well-being. This article also emphasizes the importance of addressing the needs of caregivers and the need for more research in this area. This article emphasizes the need to further investigate the effect of these treatments on the well-being of caregivers and their role in promoting communication and family resilience. Based on the findings of this study, healthcare professionals can utilize these psychological interventions to support family caregivers in clinical practice. Additionally, narrative therapy can help caregivers reframe their caregiving experiences and find new perspectives and solutions to challenges they may face. Expressive writing therapy can provide an outlet for caregivers to process their emotions and reduce stress. By incorporating these interventions into clinical practice, healthcare professionals can help caregiver’s better cope with the challenges of caregiving and improve their overall well-being. It is important for healthcare providers to recognize the unique needs of caregivers and provide support tailored to their individual circumstances. Further research in this area is needed to better understand the impact of these interventions on caregivers and their role in promoting family resilience and communication. Strengths and limitations: One of the strengths of this research was the collection of studies that examine life review interventions, and until now there has been no study of these findings that examined life review psychological interventions in a single and integrated manner. But one of the limitations of the present study is the lack of studies in this field in English and the low quality of some articles based on research evaluation criteria.
